# Anakinra for infants under six months with Kawasaki disease and coronary artery lesions: a multicenter case series and literature review

**DOI:** 10.1186/s12969-025-01143-x

**Published:** 2025-12-03

**Authors:** Giulia Inguscio, Stefano Romano, Marianna Fabi, Livia Gargiullo, Alessandra Marchesi, Maria Cristina Maggio, Maria Vincenza Mastrolia, Giovanni Battista Calabri, Gabriele Simonini, Teresa Giani

**Affiliations:** 1 Pediatric Department, School of Sciences of Human Health, University of Florence, Florence, Italy; 2 Pediatric Emergency Unit, IRCCS Azienda Ospedaliero-Universitaria Di Bologna, Bologna, Italy; 3 General Pediatric and Infectious Disease Unit, Bambino Gesù Children’s Hospital, IRCSS, Rome, Italy; 4 Pediatric Unit, Bambino Gesù Children’s Hospital, IRCCS, 00100 Rome, Italy; 5 Department of Health Promotion, Maternal and Infantile Care, Department of Internal Medicine and Medical Specialties “G. D’Alessandro”, University of Palermo, Palermo, Italy; 6 Rheumatology Unit, ERN ReCONNET Center, AOU Meyer Children’s Hospital IRCCS, Viale Pieraccini 24, Florence, 50139 Italy; 7 Cardiology Unit, Meyer Children’s Hospital IRCCS, Florence, Italy

**Keywords:** Anakinra, Coronary artery lesions, Infants, Intravenous immunoglobulin, Kawasaki disease

## Abstract

**Background:**

Infants with Kawasaki Disease (KD) have a higher risk of incomplete presentations, IVIG resistance, and coronary artery lesions (CALs). IL-1 plays a key role in the pathogenesis, highlighting its potential as a therapeutic target.

**Objective:**

To report a multicenter Italian experience and review the literature on anakinra use in KD infants with CALs.

**Methods:**

We retrospectively reviewed charts of patients aged ≤ 6 months treated with anakinra at four Italian centers between 2015 and 2024.

A systematic Literature search was also conducted in PubMed, Scopus, Embase, and the Cochrane Library up to October 2024.

**Results:**

Eight infants were included. The median age at diagnosis was 2.75 months. Six had incomplete KD. All were resistant to first-line treatment and all developed CALs, which were detected at a median of 9.5 days from fever onset. Anakinra was initiated at a median of 18 days from fever onset and 1.5 days after CALs detection. One patient received only subcutaneous anakinra. Seven infants underwent intravenous administration (median dose 8.5 mg/kg/day), four of whom received an initial bolus (median dose 2.75 mg/kg), and six were subsequently switched to subcutaneous dosing. Median total treatment duration was 22.5 days. CALs completely resolved in five patients, and improved in two. One treatment-related adverse event was reported.

The literature review identified nine additional infants ≤ 6 months; seven showed systemic improvement and five had coronary improvement, and no adverse events were reported after anakinra treatment.

**Conclusion:**

Anakinra may be a promising and well-tolerated option for infants with KD and CALs, especially in IVIG-resistant or high-risk cases. While adverse events were unusual, further studies are needed to confirm its safety and efficacy.

**Supplementary Information:**

The online version contains supplementary material available at 10.1186/s12969-025-01143-x.

## Key points

• This is the first multicenter Italian experience combined with a focused literature review on the use of anakinra in children aged ≤6 months. It offers an overview of current practices, emphasizing key aspects such as timing, dosing strategies, routes of administration, and treatment duration to optimize clinical outcomes.

• Anakinra emerges as a safe and effective treatment option for IVIG-resistant infants with Kawasaki Disease and coronary artery lesions. Early anakinra intravenous administration ensures rapid control of inflammation and improves patient comfort.

• The findings support the potential integration of IL-1 blockade into the treatment algorithm for infants with Kawasaki Disease, paving the way for future prospective studies. These will be essential to confirm these observations and establish standardized treatment protocols for this high-risk population.

**Supplementary Information:**

The online version contains supplementary material available at 10.1186/s12969-025-01143-x.

## Introduction

Kawasaki disease (KD) is an acute pediatric systemic febrile vasculitis, primarily affecting medium-sized arteries [1, 2]. Coronary artery lesions (CALs) are the typical complications, observed in up to 25% of untreated patients [3, 4]. KD is one of the most common vasculitis in children under 5 years of age and the childhood leading cause of acquired heart disease in developed countries [5]. Prominent clinical features include persistent fever, bilateral conjunctival injection, polymorphic rash, erythema of the lips and oral cavity, cervical lymphadenopathy, and extremity changes [1]. Approximately 15–35% of children [6] shows an incomplete presentation, as the minimum number of diagnostic criteria are lacking [1]. Moreover, atypical presentation may overwhelm the clinical appearance. Incomplete and atypical KD can delay diagnosis and treatment, increasing the risk of coronary complications [7, 8]. Intravenous immunoglobulins (IVIG) at 2 g/kg is the standardized first-line therapy, reducing the percentage of CALs to 5% [1, 5, 9]. However, up to 20% of treated patients are refractory, experiencing persistent or recurrent fever [10]. Several options are now available for resistant children, including a second dose of IVIG, corticosteroids, infliximab, anakinra, or calcineurin inhibitors. However, none of these are standardized or officially recommended [11].

Infants younger than six months represent a particularly vulnerable subgroup, accounting for about 10% of all KD cases. They more frequently present with incomplete or atypical features and have a higher risk of resistance to IVIG and CALs [12, 13]. A monocentric Italian study [14] found that 63% of infants under 6 months developed cardiac complications, and a large North American cohort similarly reported an increased risk of CALs in younger patients [15].

Given the growing recognition of interleukin-1 (IL-1) involvement in the inflammatory pathogenesis of KD, anakinra, an IL-1 receptor antagonist, has emerged as a potential therapeutic option. However, data on its use in very young infants are still limited.

This study reports the experience of four Italian centers administering anakinra following first-line treatment failure in infants with KD who developed cardiac involvement.

Additionally, we conducted a review of the current Literature on the use of anakinra in KD children within the first 6 months of age, focusing on its efficacy and safety.

## Methods

### Cohort analysis

We retrospectively selected KD patients from four Italian pediatric hospitals—AOU Meyer IRCCS in Florence, S. Orsola-Malpighi Hospital in Bologna, Bambino Gesù Children’s Hospital in Rome, Ospedale dei Bambini G. “Di Cristina” in Palermo—using the International Classification of Diseases, Nine Revision (ICD-9) coding for patients hospitalized between January 2015 and July 2024. Inclusion criteria were the diagnosis of KD according to the AHA criteria [4], age 6 months or younger, and treatment with anakinra. CALs were classified according to the AHA criteria [4] (ectasia: Z-score 2 to < 2.5; small aneurysm: Z-score ≥ 2.5 to < 5; medium aneurysm: Z-score ≥ 5 to < 10; giant aneurysm: Z-score ≥ 10). Cardiac involvement was used as a broader term encompassing not only coronary artery abnormalities but also other cardiovascular manifestations such as myocarditis, pericardial effusion, or impaired myocardial function.

Improvement in systemic inflammation was defined as the resolution of fever and/or a decrease in inflammatory markers.

This study was conducted as a retrospective chart review using fully anonymized data, in accordance with national regulations and institutional policies. Therefore, formal ethics committee approval was not required.

### Literature review

**Fig. 1:**
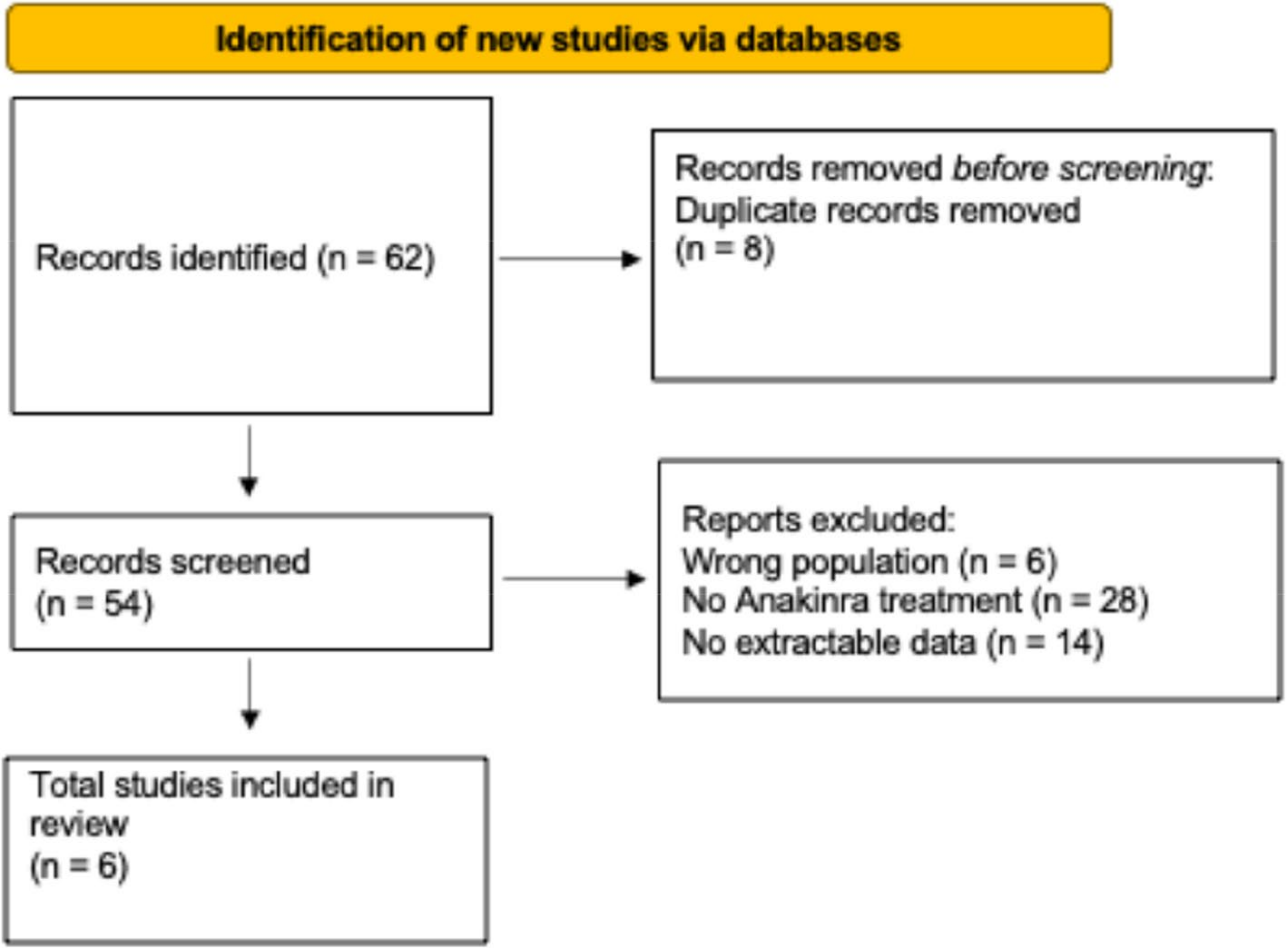
PRISMA flow diagram of study selection

We reviewed the English Literature in PubMed, Scopus, Embase, and Cochrane Library up to 1st October 2024 to identify KD infants treated with anakinra. The keywords used were: ("Kawasaki disease" OR “Kawasaki syndrome” OR "mucocutaneous lymph node syndrome" OR "Coronary artery lesions" OR "CALs") AND ("anakinra” OR “Interleukin-1 Receptor Antagonist” OR “IL-1RA”OR “Recombinant interleukin-1 receptor antagonist” OR “Anti-IL-1 therapy” OR “Interleukin-1 blockade” OR “IL-1 inhibitor") AND ("infant" OR "child" OR "pediatric") AND ("treatment outcome" OR "efficacy" OR "safety" OR "Adverse effects" OR "Side effects" OR "Tolerability" OR "Complications"). Duplicate studies were removed following the initial search. Additionally, cases involving patients older than six months, patients not treated with anakinra, and those not meeting the aforementioned criteria were excluded from the analysis. Case reports, case series and observational studies have been included. Two reviewers (GI, SR) independently evaluated the titles and abstracts to identify articles suitable for full-text review. Both reviewers assessed the entire article according to predefined inclusion and exclusion criteria. A PRISMA flow diagram (Fig. 1) was created to illustrate the study selection process.

## Results

### Results from the cohort

Eight patients met the inclusion criteria: four were from AOU Meyer IRCCS in Florence, two from S. Orsola-Malpighi Hospital in Bologna, one from Bambino Gesù Children’s Hospital in Rome, and one from G. Di Cristina Children’s Hospital in Palermo.

During the study period, the participating centers identified 504 patients with KD, including 58 infants under 7 months of age. Of these, 8 (13.8%) received anakinra. Notably, all eight were diagnosed in the last 15 months, accounting for 80% of the 10 infants under 7 months recorded during that time.

Among the patients included, three patients were female. The median age at diagnosis was 2.75 months (range: 2–5). In six of the eight cases, the disease was incomplete, showing non-exudative conjunctivitis and rash; one infant also had lip hyperemia.

The median fever duration was 8 days (range: 5–18). IVIG was administered as first-line therapy at a median of 7.5 days from fever onset (range: 4–15). The first CALs were identified at a median of 9.5 days (range: 5–32).

Anakinra was initiated at a median of 18 days from fever onset (range: 5–33; interquartile range [IQR]: 20), and 1.5 days from CALs detection (range: 0–19; IQR: 11). One patient received only SC anakinra at a dose of 6 mg/kg/day. Seven children were treated with IV anakinra (median dose 8.5 mg/kg/day); prior to continuous infusion, four of them received IV boluses with a median dose of 2.75 mg/kg (range: 2–5).

The median duration of IV anakinra was 8 days (range: 5–14), and six of the IV-treated children subsequently transitioned to SC administration at a dosage of 3 mg/kg/day (range: 2.5–8), for a total treatment duration of 22.5 days (range: 6–91). All patients received acetylsalicylic acid (ASA) at antiaggregant dosage (3–5 mg/kg/day). ASA was discontinued after 6–8 weeks in 5 out of 8 patients; the remaining 3 continued therapy due to persistent CALs.

Although the main indication for anakinra initiation in all patients was the detection or progression of CALs, six patients were febrile at the time of treatment initiation (one of whom experienced a recrudescence after a period of defervescence), and fever resolved in all cases within 2 days of treatment.

In 6 out of 8 patients, anakinra administration was followed by a reduction or normalization of C-reactive protein. Clinical general conditions improved in all patients. Complete resolution of CALs was observed in five patients, with a median time to resolution of 25.5 days (range:10–88; IQR: 52) from disease onset, while two additional patients showed a clear improvement. In the remaining patient, coronary lesions progressed despite control of systemic inflammation, and escalation of therapy.

In order to control disease activity, anakinra administration was preceded by a second dose of IVIG in five patients and by intravenous methylprednisolone (1 bolus, 2 mg/kg) in two.

Anakinra-related adverse events were reported in one out of eight patients (12.5%), consisting of diarrhea, rash flare, and hypereosinophilia (1,460/mm^3^), which appeared after 5 days of treatment and resolved promptly following drug discontinuation.

In patient 7 for persistence of increased inflammatory markers and progression of coronary aneurysms, anakinra was switched to Infliximab 5 mg/kg. Despite a progressive resolution of systemic inflammation, echocardiogram follow-up showed worsening of coronary aneurysms (LMCA 5 mm, LAD 10.8 mm, Cx 6 mm, RCA 9 mm) and rosary bead-like appearance with saccular aneurysm of the RCA and LAD. He was then transferred to a cardiac surgery centre, where terapy with cyclosporin was started, with stabilization of coronary lesions.

Long-term clinic and echocardiographic follow-up duration ranged from 6 months to four years,

with a median of 10 months.

The main demographic, clinical, laboratory, and therapeutic characteristics of the eight infants in our cohort are summarized in Table 1.

**Table 1:**
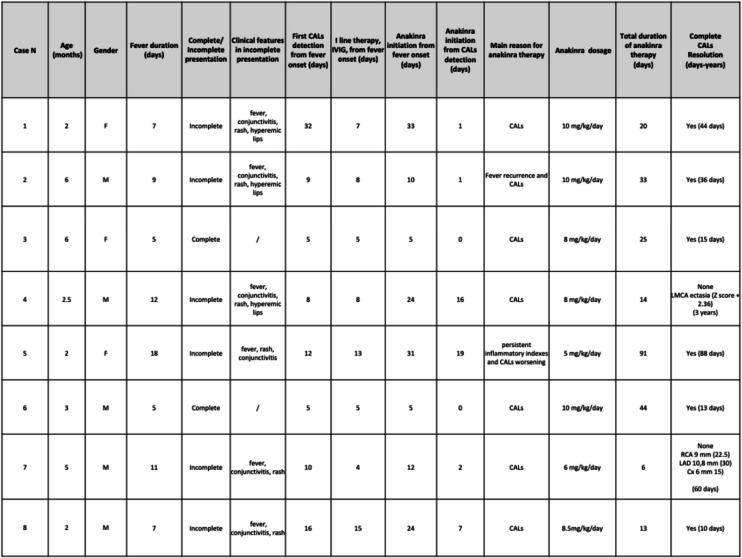
Main features of the infants with Kawasaki disease treated with anakinra in our cohort

CALs are classified according to the AHA criteria^4^ (ectasia: Z-score 2 to <2.5; small aneurysm: Z-score ≥ 2.5 to <5; medium aneurysm: Z-score ≥5 to <10; giant aneurysm: Z-score ≥10)

Abbreaviations: *M* Male, *F* Female, *Cals* Coronary artery lesions, *IVIG* Intravenous immunoglobins, *IV* Intravenous, *MAS* Macrophage activiation syndrome, *NS* Notspecified, *RCA* Right coronoray artery, *LCA* Left coronary artery, *LAD* Left anterior descending, *LMCA* Left main coronary artery, *Cx* circumflex artery

### Results from the literature review

Literature search identified 52 articles, of which 6 met the inclusion criteria. The study selection process is summarized in the PRISMA flow diagram (Fig. 1), while the main findings are presented in Supplementary Table S1b. One article is a case series [5], while the others are individual case reports [16–20]. All the patients described were infants under 7 months of age (mean age 3.8 months), including 6 males and 3 females. Five patients presented with complete KD, 3 with incomplete presentation, and 1 with an atypical form.

All infants exhibited cardiac involvement, in terms of CALs, either at diagnosis (3/9) or during follow-up (6/9), and all received IVIG as first-line therapy. Additionally, five of the nine patients received corticosteroids, and one was treated with Infliximab prior to starting Anakinra. The median time from fever onset to Anakinra treatment was 24 days (range 9–87 days) and 13 days (range 3–24) from CALs detection, with a mean starting dosage of 4.4 mg/kg/day and a median treatment duration of 58 days. Anakinra was effective in reducing systemic inflammation in 7 of the 9 patients, as evidenced by the resolution of fever and/or a decrease in inflammatory markers; moreover, Anakinra was effective in improving cardiac outcomes in 5 of the 9 patients.

In particular, in three patients Anakinra was started after fever resolution because of appearance and/or worsening of coronary aneurysms, while in five patients fever disappeared in 0–3 days after Anakinra and in one patient fever disappeared after 8 days.

No side effects or complications were reported in any of the infants.

Comprehensive data, including individual patient details, treatment course, and progression of coronary artery lesions, for both our cohort and the cases identified through the literature review are presented in Supplementary Table S1a and S1b respectively.

## Discussion

Anakinra is a biologic agent blocking the activity of both IL-1α and IL-1β, by competing for binding to the IL-1 type I receptor (IL-1RI) [21]. It was first introduced into medical practice in 2001, when the Food and Drug Administration approved it for the treatment of rheumatoid arthritis [22]. Since then, its use has progressively expanded, with additional indications and a growing number of off-label applications, including KD.

During the COVID-19 pandemic, the use of anakinra significantly increased, and the IV route became widely adopted. In hospitalized patients IV administration reduces pain from multiple injections, and, in critically ill patients, allows for rapid and stable attainment of high plasma drug concentrations [23–25]. Additionally, continuous IV anakinra infusion has proven to be both effective and safe [26–29]. Anakinra’s short half-life facilitates rapid dose adjustments and easy transitions from IV to SC administration.

Today, the indications for anakinra have broadened, and its off-label use has extended to several other conditions, including KD.

Experimental murine models have highlighted the central role of IL-1 signaling in the pathogenesis of this vasculitis and the development of CALs, with significantly increased IL-1 transcript levels in IVIG-resistant children [30, 31]. In the same models, anakinra was shown to significantly reduce the inflammatory response, suggesting potential benefits in preventing cardiovascular complications [32–35]. These findings are consistent with clinical data. In 2021, the results of the first open-label, phase IIa clinical trial (KAWAKINRA, NCT02390596) provided strong evidence for the effectiveness of early anakinra administration in patients with resistant KD, showing improvements in systemic inflammation and CALs, along with a favorable safety profile [36].

Kessel et al [37] support the rationale for IL-1 blockade with anakinra in IVIG-resistant KD, highlighting the central role of IL-1β-driven inflammation and its association with key biomarkers such as LRG1 and neutrophilia. Similarly, Armaroli et al [38] demonstrated that S100A12-driven sterile inflammation is critically mediated by monocyte-derived IL-1, and that IL-1 inhibition can prevent vascular wall damage and coronary artery remodeling, further reinforcing the rationale for IL-1 blockade.

In parallel, Anakinra has also shown safety and efficacy in other inflammatory diseases affecting children, including infants [39].

Additionally, the pharmacokinetic properties of Anakinra, such as its high subcutaneous bioavailability (95%), short half-life (4–6 h), and rapid peak concentration (3–7 h), support its adaptability in clinical practice [23].

In our multicenter experience, we treated eight Caucasian infants (mean age 2.75 months) with resistant KD and coronary artery involvement (seven with aneurysms, one with ectasia); three also had pericardial effusion.

Regarding the route**,** the IV administration was preferred in most cases.IV route was preferred in most cases, as it allowed for rapid and stable attainment of high plasma drug concentrations in these hospitalized children, while minimizing the discomfort of repeated subcutaneous injections [23–25, 40]. Continuous IV infusion has previously been shown to be safe in children with MAS or cytokine storm [26–29], and high doses (up to 15 mg/kg/day) have been well tolerated even in neonates [41, 42].

Regarding the dosing strategy, we opted to initiate treatment with higher doses, applying a step-down approach. This choice was driven by the rationale of achieving rapid inflammation control to promptly halt the progression of CALs and promote early regression and healing. This decision to start with high IV doses was based on clinical judgment and supported by our recent experience with this approach during the COVID-19 pandemic.

Following clinical stabilization**,** patients were transitioned to SC administration after 5–12 days.

In terms of outcomes**,** all patients showed rapid clinical improvement and fever defervescence, following anakinra initiation, with and normalization of inflammatory markers in the majority of the cases within a few days. Notably, five infants achieved complete regression of CALs within a median of 25 days—much earlier than the typical regression time of up to six months reported in the literature for small aneurysms [43]. In one case (patient #5), where anakinra was started later, 19 days after CALs were first detected, coronary resolution was delayed, suggesting a critical window of opportunity for anakinra achieving its optimal effect. Conversely, in patient #7, who experienced a severe inflammatory response and received anakinra only via SC administration, a progression of coronary damage was observed.

Follow-up assessments ranging from six months to one year showed stability or normalization of the coronary artery status in all patients, with no evidence of late progression. Although the short-term outcomes were encouraging, long-term data on the effects of early IL-1 blockade in infants with KD remain limited. Given the very young age of these patients and the novelty of this therapeutic approach, ongoing monitoring is warranted to better assess both the durability of vascular outcomes and the long-term safety profile of anakinra.

Compared to the literature, the effectiveness of anakinra in the nine cases previously reported appears less encouraging than the outcomes observed in our cohort. Although systemic inflammation resolved in seven of the nine infants described [5, 17–20], coronary improvement was reported in five, often without clear information on the timing or degree of regression. Notably, one patient died from a pericardial hemorrhage, likely due to aneurysm rupture shortly after treatment initiation [5], and others required escalation to anti-TNF agents due to persistent coronary involvement [17, 18, 20].

Several factors may explain these differences in outcome, including:

- Timing of treatment: First-line IVIG was administered earlier in our patients (median of 7.5 days from fever onset) compared to a median of 13.2 days in the Literature. Anakinra was also introduced earlier, at a median of 18 days from symptom onset and approximately 1 day after CALs detection, whereas in the Literature it was started at a median of 24 days from disease onset and 13 days after CALs detection.
- Initial anakinra dosing: The mean starting dose in our cohort was higher (8.5 mg/kg/day) compared to 4.4 mg/kg/day in the literature, potentially contributing to faster control of inflammation and regression of coronary lesions.
- Route of anakinra administration: In our series, IV administration was preferred, allowing for rapid and stable drug levels. In contrast, the route of administration was often unspecified [19] in published cases, and when reported, was commonly SC.

Moreover, several infants described in the literature presented with an aggressive disease. Three were diagnosed with macrophage activation syndrome (MAS) [18, 19, 21], two with KD shock syndrome [5], and four developed giant coronary aneurysms [5, 17, 21].

Regarding safety, a single treatment-related adverse event was observed in our cohort: a 2-month-old male infant developed a DRESS (Drug Reaction with Eosinophilia and Systemic Symptoms)-like reaction, which promptly resolved upon anakinra discontinuation. No other adverse events were reported in our cohort or in the literature.

The only reported KD relapse [19] in the literature, was not associated with a cardiac deterioration.

Finally, with regard to long-term outcomes, follow-up assessments ranging from six months to four years demonstrated either complete normalization or, at minimum, stabilization of CALs in all patients, with no evidence of late progression. While the short-term outcomes following early IL-1 blockade were encouraging, data on its long-term impact in infants with KD remain scarce. Given both the very young age of these patients and the novelty of this therapeutic approach, ongoing clinical monitoring is essential to better understand the durability of vascular responses and the long-term safety profile of anakinra.

This study has several limitations that must be acknowledged. First, its retrospective design inherently limits the ability to draw causal inferences and may be subject to selection and reporting biases. Second, the small sample size, though the largest reported in this specific population, reduces the statistical power and generalizability of our findings. Third, the absence of a control group receiving standard therapy alone makes it difficult to isolate the specific effect of anakinra. Additionally, variation in treatment regimens across patients, including differences in dosage, route of administration, timing, and use of adjunctive therapies such as corticosteroids or additional IVIG, introduces heterogeneity that may influence outcomes. The relatively short follow-up for some patients may also limit our ability to fully assess long-term cardiac outcomes, particularly regarding persistent or regressing coronary artery lesions.

Despite these limitations, our results suggest a potential benefit of early IL-1 blockade with anakinra in infants with KD and coronary involvement, particularly when IVIG fails. Future prospective, multicenter studies with standardized treatment protocols, longer follow-up, and larger sample sizes are essential to confirm these observations. Moreover, randomized controlled trials would be crucial to establish the efficacy and safety of anakinra in comparison to other second-line treatments, and to define optimal timing, dosage, and mode of administration in this high-risk population.

## Conclusions

Anakinra may be a promising therapeutic option for patients with KD who are resistant to first-line treatment, emerging as an effective and safe drug even in very young infants, including those aged six months or younger. Early administration offers rapid resolution of fever and inflammatory markers and plays a crucial role in stabilizing and normalizing CALs, which is essential for long-term outcomes. Moreover, its manageable and safe profile allows for increased dosages, which may be critical to achieving optimal therapeutic effects. The IV route has proven to be well-tolerated and facilitates the rapid attainment of effective plasma concentrations, while minimizing discomfort in hospitalized children. It also allows for a smooth transition to SC administration, once the clinical course stabilizes.

These preliminary findings support the integration of IL-1 blockade into the treatment algorithm of high-risk KD infants, and call for structured validation through prospective trials.

## Supplementary Information

Supplementary Material 1.

## Data Availability

The datasets generated and/or analyzed during the current study are not publicly available due to patient confidentiality and institutional restrictions, but are available from the corresponding author on reasonable request.
